# Grapefruit Flavonoid Naringenin Sex-Dependently Modulates Action Potential in an In Silico Human Ventricular Cardiomyocyte Model

**DOI:** 10.3390/antiox11091672

**Published:** 2022-08-27

**Authors:** Henry Sutanto, Decsa Medika Hertanto, Hendri Susilo, Citrawati Dyah Kencono Wungu

**Affiliations:** 1 Department of Internal Medicine, Faculty of Medicine, Universitas Airlangga, Surabaya 60131, Indonesia; 2 Department of Internal Medicine, Dr. Soetomo General Hospital, Surabaya 60286, Indonesia; 3 Department of Cardiology and Vascular Medicine, Universitas Airlangga Teaching Hospital, Surabaya 60115, Indonesia; 4 Department of Physiology and Medical Biochemistry, Faculty of Medicine, Universitas Airlangga, Surabaya 60131, Indonesia

**Keywords:** flavonoid, citrus fruit, grapefruit juice, naringenin, antioxidant, cardiovascular risk, cardiac arrhythmia, ion channel electrophysiology, computational modeling

## Abstract

Recent in vitro studies showed that grapefruit (*Citrus* × *paradisi*) flavonoid naringenin alters the function of cardiac ion channels. Here, we explored the effect of naringenin on cardiomyocyte action potentials (APs) using a detailed in silico model of ventricular electrophysiology. Concentration-dependent effects of naringenin on seven major cardiac ion channels were incorporated into the Tomek–Rodriguez modification of O’Hara–Rudy (ToR-ORd) human ventricular endocardium model. To investigate the sex-dependent effect of naringenin, previously reported sex-specific ionic modifications were implemented into the model. Next, populations of 1000 models accommodating intercellular variability were generated. The results show, naringenin at various concentrations prolonged AP duration (APD) in male and female cardiomyocytes. Pacing cells at higher frequencies abbreviated APD differently in males versus females; for example, at 3 Hz, 50 μM naringenin induced AP and calcium alternans only in the female cardiomyocyte. Finally, a population modeling approach corroborated that naringenin significantly prolonged APD in a concentration-dependent manner, with a larger effect in females than in males. In conclusion, our study demonstrates that the APD-prolonging effect of naringenin was larger in females, and that pacing at faster rates induces AP alternation earlier in females, suggesting a potentially higher proarrhythmic risk of naringenin in females than in males.

## 1. Introduction

In addition to its high nutritional content (e.g., carbohydrate, dietary fiber, vitamin B6, vitamin C, potassium, magnesium, and calcium), grapefruit (*Citrus* × *paradisi*) also contains several bioactive phytochemicals, such as carotenoids, limonoids, flavonoids, and furanocoumarins, which are believed to be beneficial against diseases and pathological conditions [1,2]. Previous studies demonstrated that furanocoumarins (e.g., bergamottin, paradisin, bergapten, epoxy-bergamottin, and 6,7-dihydroxybergamottin) primarily exhibit antioxidative, anti-inflammatory, antimicrobial, anticancer, and neuro- and osteoprotective activities [1]. For example, furanocoumarins interfere with several biological pathways, modulating autophagy, apoptosis, angiogenesis, and metastatic processes via the alteration of nuclear factor κB (NF-κB), phosphatidylinositol-3-kinase (PI3K), Akt, p53, molecular target of rapamycin (mTOR), Janus kinase (JAK), signal transducer and activator of transcription 3 (STAT3), B-cell lymphoma 2 (Bcl-2), and vascular endothelial growth factor (VEGF) [3]. Similarly, grapefruit-derived flavonoids (e.g., naringenin) also have antioxidant, anticancer, antimicrobial, anti-inflammatory, antiadipogenic, and cardioprotective effects [2]. Numerous in vitro and in vivo studies have shown the effectivity of naringenin to protect against diseases, including asthma, Alzheimer’s disease, seizure, viral hepatitis, mosquito-borne infections, diabetes mellitus, rheumatic and inflammatory diseases, stroke, and cardiovascular and metabolic diseases [2]. Among other effects on cardiovascular system [4], naringenin has been demonstrated to protect against myocardial ischemia–reperfusion (MI/R) injury by reducing oxidative stress and endoplasmic reticulum (ER) stress via cyclic guanosine monophosphate–protein kinase G (cGMP-PKGIα) signaling [5]. Additionally, it reduces the atherogenic index of plasma value, serum non-HDL-C levels, total cholesterol/high-density lipoprotein cholesterol (HDL-C), triglyceride/HDL-C, low-density lipoprotein cholesterol/HDL-C and non-HDL-C/HDL-C ratios, as well as reduces the body mass index (BMI) and visceral fat in non-alcoholic fatty liver disease (NAFLD) [6]. Consistently, an observational study using data from the 2003–2008 National Health and Nutrition Examination Survey (NHANES) reported that among women, grapefruit consumption was associated with lower body weight and BMI, as well as plasma triglycerides and C-reactive protein [7].

Nevertheless, despite the evident benefits of grapefruit consumption, it has been widely reported that grapefruit, primarily due to its furanocoumarins content, may impair the pharmacokinetics of several drugs. Cytochrome P450 (CYP) is involved in the biosynthesis of furanocoumarins, potentially interacting with the CYP-mediated drug metabolism if consumed concurrently [1]. The most renowned drug–grapefruit interaction is facilitated by CYP3A4. This cytochrome plays a role in the metabolism of about 50% of all drugs, and is naturally located in the epithelial linings of intestines and colon, as well as in the hepatocytes. The irreversible binding of CYP3A4 with furanocoumarins reduces the amount of available enzymes significantly, thus altering the bioavailability of associated drugs. Pharmacological agents that are involved in this process include chemotherapeutic drugs, antibiotics and antimicrobials, lipid-lowering drugs, cardiovascular drugs, central nervous system drugs, immunosuppressants, and urinary tract medications [8]. The increased bioavailability of these drugs consequentially increases the risk of toxicity, and as a consequence, several serious adverse events, including life-threatening arrhythmias, rhabdomyolysis, and renal failure, have been documented following the consumption of grapefruit, together with CYP3A4-associated drugs [8].

In addition, previous studies have also demonstrated that naringenin, a bioactive flavonoid of grapefruit and grapefruit juice, could affect cardiac electrophysiology through the alteration of cardiac ion channel function. For example, Scholz et al. [9] used *Xenopus laevis* oocytes to investigate the electrophysiological properties of the human ether-à-go-go-related gene (hERG) inhibition by naringenin. They found that both the open and inactivated states of the channel were inhibited by naringenin, with a 50% inhibitory concentration (IC_50_) of 102.6 μM [9]. Similarly, among other flavonoids tested, Zitron et al. [10] discovered that naringenin substantially inhibits the hERG channel in heterologously expressed cells, with IC_50_ in *Xenopus laevis* oocytes of 102.3 μM and in human embryonic kidney (HEK) cells of 36.5 μM. Another in vitro study using *Xenopus laevis* oocytes also reported the additive hERG-inhibitory effect of naringenin and several rapid delayed-rectifier potassium current (I_Kr_)-blocking antiarrhythmic drugs [11]. Recently, Sanson et al. [12] explored the effect of naringenin on seven major cardiac ion channels commonly used for the comprehensive in vitro proarrhythmia assay (CiPA). They employed HEK and Chinese hamster ovary (CHO) cells, and demonstrated that all the CiPA ion channels were dose-dependently inhibited by naringenin [12].

Yet, although those earlier studies have shed some light on the ionic consequences of naringenin, at present, the effect of naringenin on cardiac action potential (AP) and arrhythmogenesis is unknown. Thus, in this in silico study, we sought to investigate the electrophysiological effect of various concentrations of naringenin on human ventricular APs, and evaluate the role of sex in modulating such an effect.

## 2. Materials and Methods

**Figure 1:**
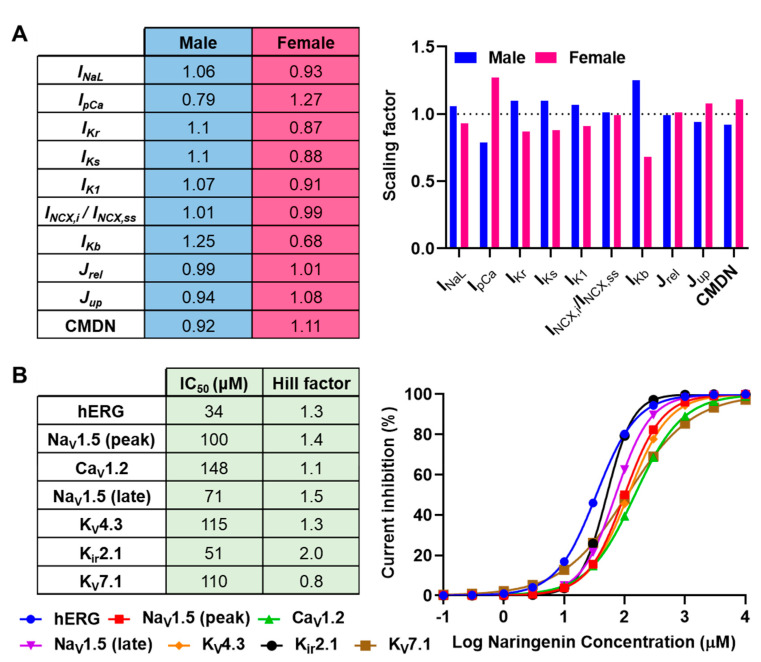
Sex-specific ionic modifications implemented in the human ventricular cardiomyocyte model and concentration-dependent inhibitions of cardiac ion channels by naringenin. (**A**) Ten Tomek–Rodriguez adaptations of O’Hara–Rudy (ToR-ORd) model parameters were modified to obtain sex-specific human ventricular endocardium electrophysiology. The scaling factors were obtained from Peirlinck, Sahli Costabal, and Kuhl [14]. (**B**) The concentration–response curves displaying the concentration-dependent inhibition of seven major cardiac ion channels together with the associated 50% inhibitory concentrations (IC_50_) and Hill factors [12].

We employed the Tomek–Rodriguez modification of O’Hara–Rudy (ToR-ORd) human ventricular cardiomyocyte model [13] to simulate the effect of naringenin on ventricular cardiomyocyte electrophysiology. The endocardial cell type was specifically employed for this study. The ToR-ORd model was chosen because it is a well calibrated and independently validated model of human ventricular cardiomyocyte that enables in silico analyses of both healthy and disease conditions, with or without the presence of pharmacological inhibition of ion channels [13]. Subsequently, we incorporated sex-specific ionic modifications described by Peirlinck et al. [14] into the baseline model to obtain the male and female cell phenotypes. In particular, modifications to 10 model parameters, consisted of ionic current conductance, fluxes, and calcium-handling parameters (i.e., late sodium current (I_NaL_), sarcolemmal calcium pump current (I_pCa_), I_Kr_, slow delayed-rectifier potassium current (I_Ks_), inward-rectifier potassium current (I_K1_), ratio between intracellular and subsarcolemmal sodium–calcium exchange current (I_NCX,i_/I_NCX,ss_), background potassium current (I_Kb_), sarcoplasmic reticulum (SR) calcium release flux (J_rel_), SR calcium uptake flux (J_up_) and calcium buffering capacity through calmodulin concentration (CMDN)), were added to the baseline ToR-ORd model to create the sex-specific model phenotypes (Figure 1A).

Next, concentration-dependent inhibitions of seven cardiac ion channels (i.e., fast sodium current (I_Na_) and I_NaL_-linked Na_V_1.5, L-type calcium current (I_CaL_)-linked Ca_V_1.2, transient-outward potassium current (I_to_)-linked K_V_4.3, I_K1_-linked K_ir_2.1, I_Kr_-linked hERG, and I_Ks_-linked K_V_7.1) by naringenin [12] were incorporated into the model (Figure 1B) by tuning the maximum conductance of the associated currents in the ToR-ORd model. The Hill equation used to generate the concentration–response curves in Figure 1B (right panel) is as follows:

$$\mathrm{Current}\;\mathrm{inhibition}\;\left(\%\right)=100-\frac{100}{1+{\left(\frac{\mathrm{naringenin}\;\mathrm{concentration}}{{\mathrm{IC}}_{50}}\right)}^{\mathrm{Hill}\;\mathrm{factor}}}$$

To assess potential consequences of intercellular variability on the electrophysiological effect of naringenin, the maximum conductance of nine major ionic currents (I_Na_, I_NaL_, I_CaL_, I_Kr_, I_Ks_, I_K1_, I_to_, I_NCX_, and the sodium–potassium pump current (I_NaK_)) were scaled based on a normal distribution with mean 1.0 and standard deviation 0.2 to create populations of models, as previously conducted [15]. All simulations were performed in Myokit [16] and all results are presented during steady-state pacing at the indicated pacing frequencies (after 1000 beats of pre-pacing). Statistical analyses of more than two independent groups were performed using one-way analysis of variance (ANOVA) with Tukey’s multiple comparison test in GraphPad Prism 7 software (San Diego, CA, USA) and statistical significance is reached if *p*-value < 0.05. The model code is available at www.github.com/henrysutanto (accessed on 11 July 2022).

## 3. Results

### 3.1. Naringenin Sex-Dependently Prolongs APD in a Human Ventricular Cardiomyocyte Model

**Figure 2:**
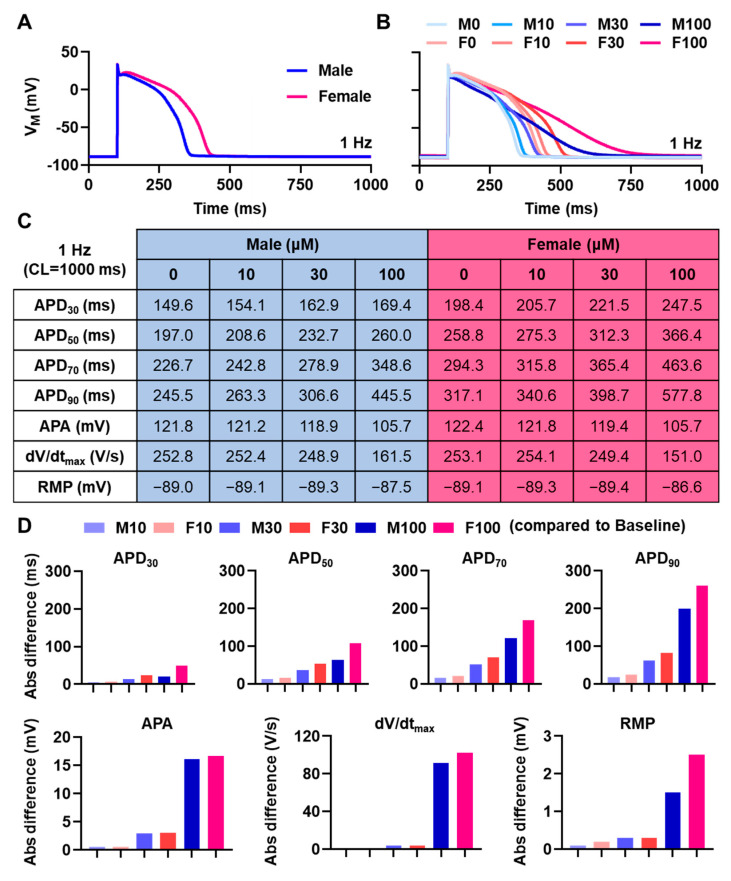
Sex-dependent effect of naringenin on the action potential of ventricular cardiomyocytes. (**A**) The comparison of male and female action potentials of ventricular endocardium. (**B**–**D**) The concentration-dependent effect of naringenin on male and female ventricular action potentials. The baseline/no-compound conditions (M0 and F0) were used to calculate the absolute (Abs) difference in (**D**). All simulations were performed in 1 Hz pacing frequency. (APA = action potential amplitude; APD = action potential duration; CL = cycle length; F0 = Female 0 μM; F10 = Female 10 μM; F30 = Female 30 μM; F100 = Female 100 μM; M0 = Male 0 μM; M10 = Male 10 μM; M30 = Male 30 μM; M100 = Male 100 μM; RMP = resting membrane potential).

As displayed in Figure 2A, the male baseline AP was substantially shorter (APD at 90% repolarization (APD_90_) = 245.5 ms) than the female baseline AP (APD_90_ = 317.1 ms), with an absolute APD_90_ difference of 71.6 ms. The administration of various concentrations of naringenin (i.e., 10, 30, and 100 μM) prolonged APD in both male and female cardiomyocytes (Figure 2B). The detailed changes in AP properties following naringenin application are shown in Figure 2C. In brief, all tested naringenin concentrations extended the APD at 30%, 50%, and 70% repolarization (APD_30_, APD_50_, and APD_70_, respectively), as well as APD_90_ in both male and female cardiomyocytes in a concentration-dependent manner. APs treated with higher concentrations of naringenin also exhibited a shorter AP amplitude (APA) and a lower upstroke velocity (dV/dt_max_) in both male and female cardiomyocytes. Additionally, 10 and 30 μM naringenin trivially hyperpolarized resting membrane potential (RMP), whereas 100 μM naringenin depolarized RMP in both sexes (−87.5 mV in males and −86.6 mV in females).

Figure 2D presents the absolute differences of AP properties as compared to the baseline values (i.e., in males with 0 μM naringenin (M0) and females with 0 μM naringenin (F0)). Naringenin induced a larger prolongation of APD_30_, APD_50_, APD_70_, and APD_90_ in female than in male cardiomyocytes. Such sex-specific APD changes were augmented in higher concentrations of naringenin. For example, at 10 μM, naringenin prolonged APD_90_ in males by 17.8 ms, whereas in females, naringenin prolonged APD_90_ by 23.5 ms (absolute difference of 5.7 ms). Furthermore, at 100 μM, naringenin prolonged APD_90_ by 200.0 ms in males and 260.7 ms in females (absolute difference of 60.7 ms). Consistently, at higher concentrations of naringenin (e.g., 100 μM), the RMP difference in females was larger than in males (2.5 mV and 1.5 mV, respectively), although in moderate concentrations, the sex-specific difference was negligible. Additionally, no noticeable difference on APA and dV/dt_max_ was documented between male and female cardiomyocytes, although a concentration-dependent effect was clearly observed, especially in 100 μM naringenin. Overall, our data in Figure 2D suggest that naringenin exhibits both sex- and concentration-dependent APD prolongation.

### 3.2. Female Cardiomyocytes Exhibit AP and Calcium Alternans Earlier at Higher Pacing Frequencies

**Figure 3:**
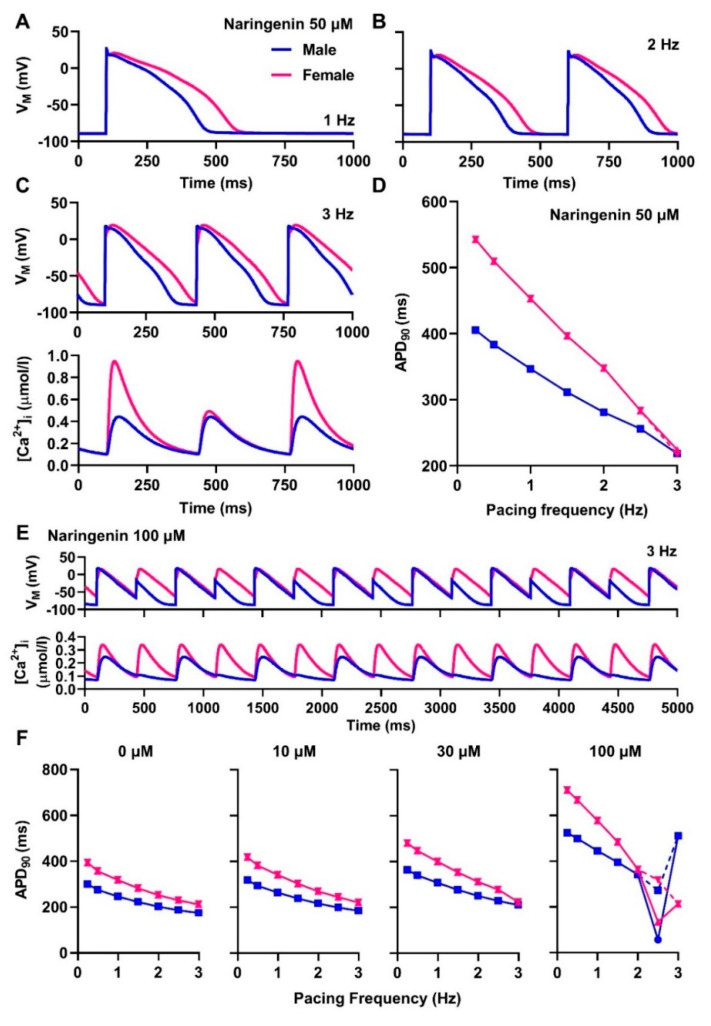
The rate-dependent effect of naringenin on human ventricular action potentials. (**A**–**C**) The rate-dependent effects of 50 μM naringenin on male and female action potentials. At 3 Hz pacing frequency (**C**), both calcium and action potential (AP) alternans were documented in the female AP, but not in the male AP. (**D**) The frequency–APD curve displaying the relationship between pacing rate and changes in AP duration at 90% repolarization (APD_90_) in response to the administration of 50 μM naringenin. (**E**) The effect of 100 μM naringenin at 3 Hz pacing frequency. (**F**) The frequency–APD curve displaying the relationship between pacing rate and changes in APD_90_ in response to the administration of other concentrations of naringenin.

Pacing cardiomyocytes at different pacing frequencies (Figure 3) revealed distinct APD-rate dependence in male vs. female cardiomyocytes. In the absence and presence of naringenin, female cardiomyocytes displayed a larger abbreviation of APD_90_ following an increased pacing rate (Figure 3D,F). With 50 μM naringenin (Figure 3A–D), in addition to the rate-dependent APD_90_ shortening, AP and calcium alternans were only observed in female cardiomyocytes at pacing frequencies of 3 Hz (basic cycle length (BCL) = 333 ms) or more. Meanwhile, neither AP nor calcium alternans was documented following 0 to 30 μM naringenin in both sexes (Figure 3F). With 100 μM naringenin, AP alternation was observed at high pacing rates, with some episodes of discordant AP and calcium alternans (Figure 3E).

### 3.3. Population Modeling Corroborates Concentration-Dependent AP Prolongation Induced by Naringenin

**Figure 4:**
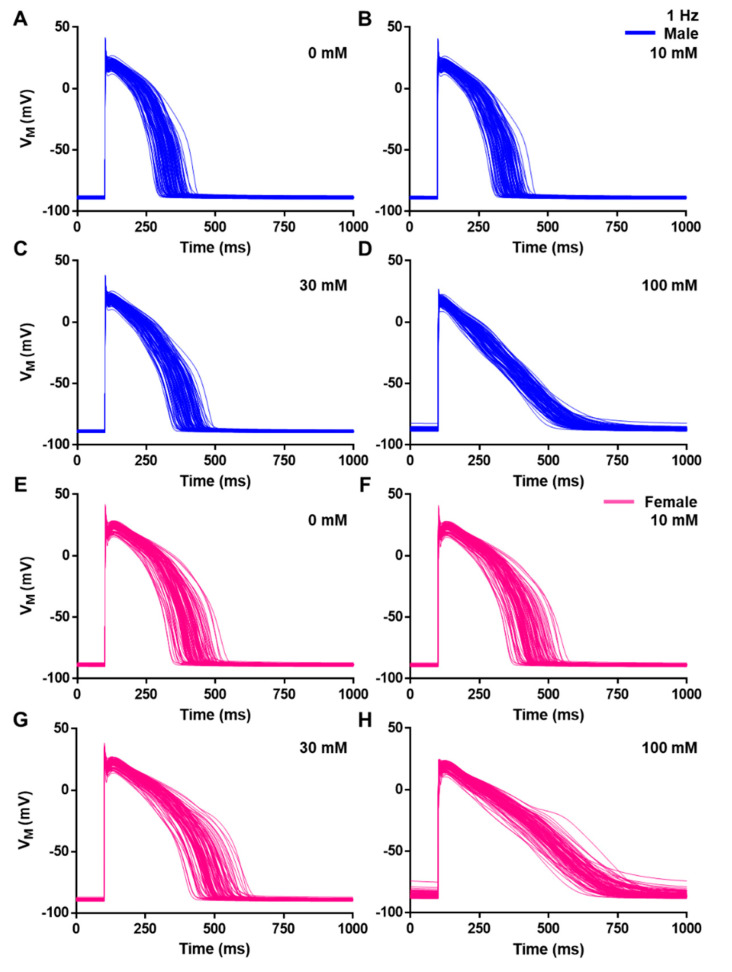
Populations of models depicting the effect of naringenin on ventricular action potentials. One thousand model variations of the ToR-ORd model were made by altering the maximum conductance of nine major cardiac ion channels to represent biological variations (e.g., intercellular variability) in ventricular cardiomyocytes. In this figure, ‘only’ 100 AP variations were shown to maintain visibility. Naringenin was administered in three different concentrations: 10 (**B**,**F**), 30 (**C**,**G**), and 100 μM (**D**,**H**). Male = blue APs (**A–D**); Female = magenta APs (**E–H**).

**Figure 5:**
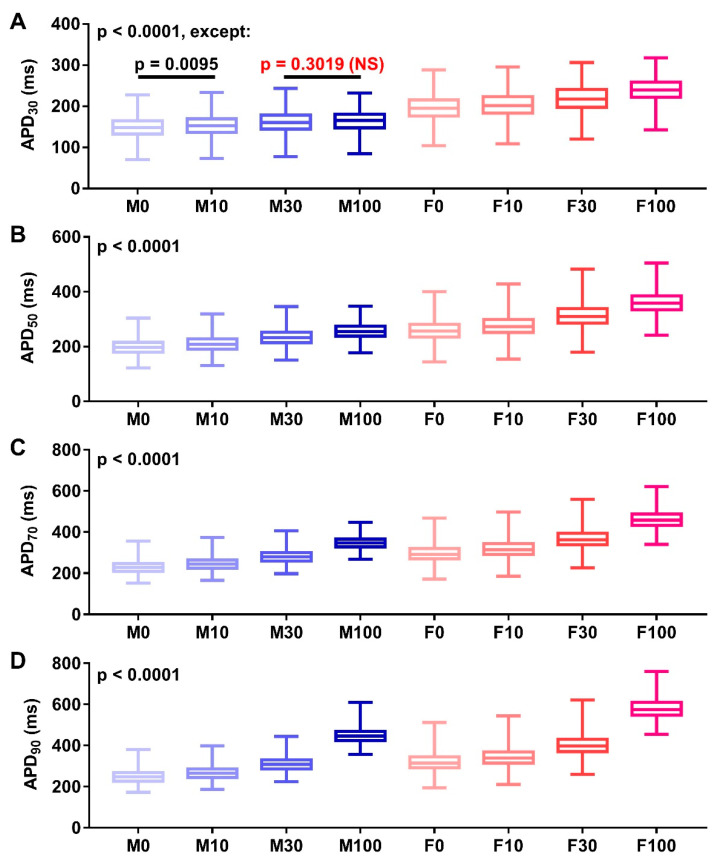
The comparison of action potential durations (APDs; APD_30_ (**A**), APD_50_ (**B**), APD_70_ (**C**) and APD_90_ (**D**)) for various concentrations of naringenin. The data were extracted from the population of models consisting 1000 model variations shown in Figure 4. Statistical analyses were performed using one-way analysis of variance (ANOVA) with Tukey’s multiple comparison test between concentrations in males and females, and in the same concentrations across sex (i.e., M0 vs. M10, M0 vs. M30, M0 vs. M100, M10 vs. M30, M10 vs. M100, M30 vs. M100, F0 vs. F10, F0 vs. F30, F0 vs. F100, F10 vs. F30, F10 vs. F100, F30 vs. F100, M0 vs. F0, M10 vs. F10, M30 vs. F30, and M100 vs. F100). Statistical significance is reached if *p* < 0.05.

To evaluate the robustness of our earlier findings in the presence of intercellular variability, we generated populations of models consisted of 1000 variations of the ToR-ORd model for each sex and applied naringenin at various concentrations (10, 30, and 100 μM) (Figure 4). The comparisons of the APD_30_, APD_50_, APD_70_, and APD_90_ derived from the populations are displayed in Figure 5. As shown in the figure, increasing the concentration of naringenin substantially prolonged APD_30_, APD_50_, APD_70_, and APD_90_ in a concentration-dependent manner, with the largest APD prolongation in 100 μM naringenin (i.e., M100 and F100). Of note, the APD differences between groups were statistically significant, except for APD_30_ at M30 vs. M100 (*p* = 0.3019; Figure 5A). Such an APD-prolonging effect of naringenin was visible in both male and female cardiomyocytes, with a significantly larger effect in females than in males, signifying the sex-dependent effect of naringenin.

**Figure 6:**
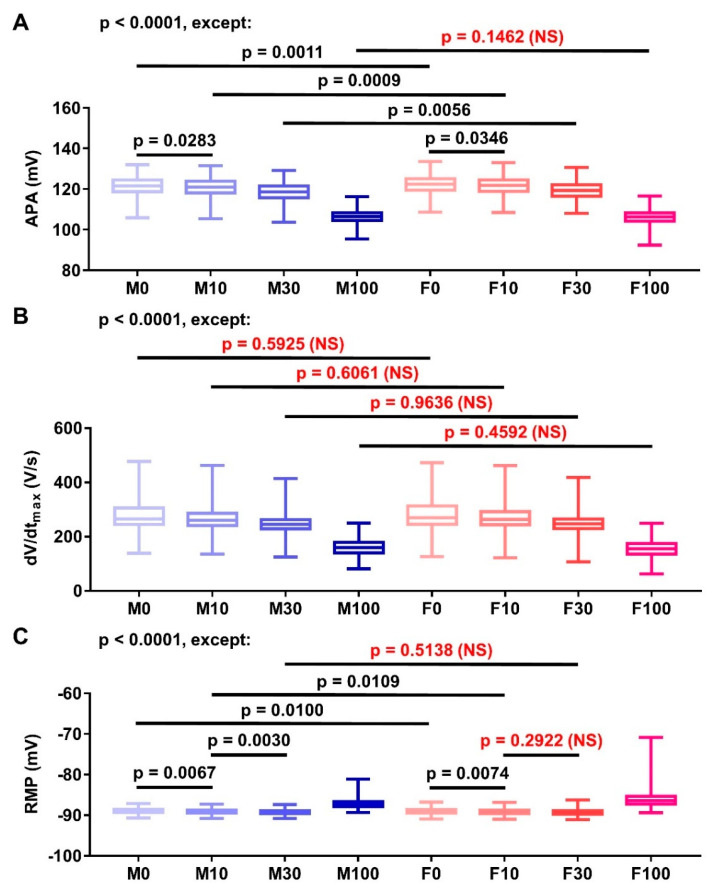
The comparison of other action potential properties (i.e., APA (**A**), dV/dt_max_ (**B**) and RMP (**C**)) in various concentrations of naringenin. The data were extracted from the population of models consisting 1000 model variations shown in Figure 4. Statistical analyses were performed using one-way analysis of variance (ANOVA) with Tukey’s multiple comparison test between concentrations in males and females, and in the same concentrations across sex (i.e., M0 vs. M10, M0 vs. M30, M0 vs. M100, M10 vs. M30, M10 vs. M100, M30 vs. M100, F0 vs. F10, F0 vs. F30, F0 vs. F100, F10 vs. F30, F10 vs. F100, F30 vs. F100, M0 vs. F0, M10 vs. F10, M30 vs. F30, and M100 vs. F100). Statistical significance is reached if *p* < 0.05. (APA = action potential amplitude; NS = not significant; RMP = resting membrane potential).

Next, the comparisons of other AP properties (i.e., APA, dV/dt_max_, and RMP) derived from the populations are depicted in Figure 6. Overall, the APA differences between groups were statistically significant, except for the comparison between the highest tested concentration (i.e., M100 vs. F100; *p* = 0.1462; Figure 6A). Meanwhile, the dV/dt_max_ differences between groups were only statistically significant within the same sex (Figure 6B). These data indicate that sex-dependent effect of naringenin on AP upstroke velocity was absent in our simulations, while the concentration-dependent effect remained present. Finally, despite the small changes in RMP, the population modeling approach still achieved statistical significance (Figure 6C), except for the RMP differences between M30 vs. F30 (*p* = 0.5138) and F10 vs. F30 (*p* = 0.2922).

## 4. Discussion

In this study, we explored the electrophysiological consequences of grapefruit flavonoid naringenin on human ventricular action potentials using an in silico approach. First, our data suggest that naringenin exhibited an APD-prolonging effect that was positively associated with its cellular concentration. Second, female cardiomyocytes displayed a larger naringenin-induced APD prolongation than male cardiomyocytes, especially for the higher concentrations of naringenin. Third, naringenin provoked AP and calcium alternans at high pacing frequencies earlier in female than in male cardiomyocytes, suggesting its possible sex-dependent proarrhythmia. Finally, we corroborated our earlier results using a population modeling approach, and showed that in the presence of intercellular variability, concentration- and sex-dependent effects of naringenin remained present, with the largest APD prolongation in females and in higher concentrations.

To the best of our knowledge, this study was the first to unravel the effect of various concentrations of naringenin on human ventricular action potentials. The APD prolongation identified in this study was consistent with clinical observations reporting the QT interval prolongation following consumption of grapefruit and grapefruit juice. For example, an open-label randomized clinical study involving 30 healthy individuals and 10 patients with congenital long QT syndrome (LQTS) reported that following the consumption of 2 L of grapefruit juice, significant prolongation of rate-corrected QT (QTc) interval was observed in both healthy subjects and LQTS patients. Importantly, the QT-prolonging effect of grapefruit juice was greater in female, as well as in LQTS patients [17]. This is consistent with our finding, showing that naringenin induced a larger APD prolongation in female than in male cardiomyocytes (Figure 2D). Similarly, another study administered 1 L of pink grapefruit juice, which is rich of naringenin (>1000 μM/L), to 10 healthy subjects and documented a significant QTc prolongation a few hours after consumption [10]. Furthermore, QTc interval prolongation and an increased QT variability index were also observed after the administration of 1 L of freshly squeezed pink grapefruit juice (with ±1400 μM/L naringenin) in 32 subjects, including 10 patients with dilated cardiomyopathy and 12 patients with hypertensive cardiomyopathy. They also showed that pink grapefruit juice impaired the temporal dispersion of cardiac repolarization in all subjects (both healthy and diseased patients), warranting further investigations on the exact pathomechanisms [18].

Next, the presence of the QT-prolonging effect of grapefruit juice, in particular naringenin, has strong clinical relevance. As explicated in the introduction section, the furanocoumarins content of grapefruit commonly interacts with drugs metabolized by CYP3A4. As the consequence, the bioavailability of certain drugs (e.g., drugs that may induce QT prolongation and exacerbate arrhythmia in the high dose) is increased, predisposing to a higher risk of drug-induced toxicity. Since our analysis, as well as other previous studies, showed that the naringenin content of grapefruit could induce APD and QT interval prolongation, excessive consumption of grapefruit and grapefruit juice together with those drugs might notably increase the duration of cardiac repolarization (e.g., marked by APD and QT interval). Such an extensive prolongation could increase the propensity of malignant arrhythmias, including Torsade de Pointes. Indeed, incidences of ventricular arrhythmias, including Torsade de Pointes, following regular consumption of grapefruit juice and in-hospital amiodarone in a 83-year-old female with atrial fibrillation were previously documented [19].

Overall, the abovementioned experimental and clinical studies, together with the current in silico study, suggest that in addition to its high antioxidant content [20,21], *Citrus* × *paradisi* also confers an ion-channel-modulating property that could alter cardiac electrophysiology in specific populations (e.g., females or people with pre-existing cardiovascular diseases). It has been known for decades that the antioxidants in citrus juices have abundant cardiovascular benefits [22]. Not only the vitamin antioxidants, ascorbic acid, tocopherol, and β-carotene were responsible for such a cardioprotective effect, but also the polyphenols and flavonoids contents in both the fruit and the juice. Some polyphenols, such as naringin and hesperetin, also exhibit a potent hypolipemic activity. They were found to facilitate the reduction in blood cholesterol and inhibit the progression of atherosclerosis in animal models [22]. In this study, we, for the first time, show that naringenin, a flavonoid content in grapefruit and grapefruit juice, could also sex-dependently modulate cardiomyocyte AP. Nonetheless, whether naringenin directly targets those cardiac ion channels structure or indirectly affects ion channels through the reduction in oxidative stress is unknown. We previously illustrated that there is a close tie between inflammation, oxidative stress, the release of reactive oxygen species, ion channel remodeling and cardiac arrhythmias [15]. Therefore, it could be that the naringenin-induced ionic remodeling was a result of this complex intertwining process and further experimental studies are warranted to elucidate the exact mechanisms.

This study has several challenges. First, we used cellular concentrations of naringenin, which are expected to be much lower than the administered dose of naringenin or the actual content of naringenin in a grapefruit due to the pharmacokinetics of naringenin. Previous studies demonstrated that despite its fast absorption, naringenin exhibits very low bioavailability due to an extensive first-pass effect in the intestine. It undergoes rapid hepatic first-pass metabolism and is transformed into glucuronide intermediary products, resulting in its limited bioavailability in plasma. After oral intake of naringin or grapefruit juice, significant concentrations of naringenin have been found in peripheral blood and urine [23], suggesting its rapid distribution and clearance. Computationally, it is currently challenging to correlate these cellular concentrations to the clinically relevant doses due to the unavailability of a coupled pharmacokinetics–cellular electrophysiological model, making it difficult to evaluate the electrophysiological effect of naringenin at such clinically relevant doses. Nonetheless, pharmacokinetics models exist, and can be implemented in the future. Second, the naringenin-induced inhibition of CiPA ion channels used in this study was measured in heterologously expressed cells, which could provide different results from human ventricular cardiomyocytes. Immortalized human cardiomyocytes exist [24], and may be used in the future to confirm previously reported concentration-dependent inhibition of cardiac ion channel by naringenin [25].

## 5. Conclusions

Our in silico study shows that grapefruit and grapefruit juice flavonoid naringenin prolonged human ventricular APD in a concentration-dependent manner. The APD prolongation was larger in female than in male cardiomyocytes, particularly at high naringenin concentrations. Moreover, at high pacing rates, naringenin induces AP and calcium alternans earlier in females than in males, suggesting its higher proarrhythmic risk in females, consistent with a previous clinical trial. In general, due to its potential harm, grapefruit and grapefruit juice consumption should be moderated. Further research is needed to experimentally confirm the accuracy of our in silico prediction, and to investigate the exact cardiac consequences of low and moderate doses of grapefruit juice, especially in people with pre-existing cardiovascular diseases.

## Data Availability

The data are contained within the article. The model code is available at www.github.com/henrysutanto (accessed on 11 July 2022).
